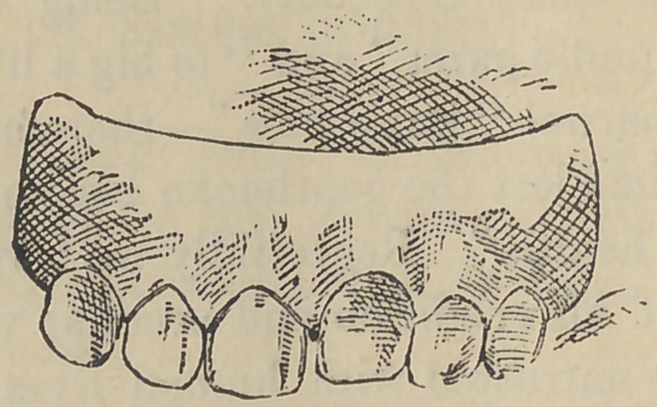# An Interesting Case

**Published:** 1889-08

**Authors:** 


### EDITORIAL.

### An Interesting Case.

The following came to hand a few days ago.

SOUTH BEND, INDIANA.

EDITOR OF THE REGISTER,

*Dear Sir:*—Please give me advice in the following case: Mrs. W., aged twenty-five, had seven years ago a recession of the left central incisor, or rather, a shortening of the tooth of fully a line in which the tooth settled into the socket; this was done suddenly or within a few days, and without any accident or appreciable disease; no soreness or pain either at the time or afterwards; the other teeth in the anterior part of the mouth were all sound and in good position. The root of a first bicuspid I extracted a short time ago. I knew the person when her teeth were perfectly regular. I am at a loss as to the cause of this affection. How could it have occurred? And especially since no accident or disease were recognized as being present or influincing the case. The patient desires a remedy; in what way shall or can it be accomplished? I enclose a cast of the case which shows exactly the condition. It will be seen that the margin of the gum has receded with the tooth so that it presents as great anterior surface as its neighbor. Please give me your suggestions as to the best method of meeting the difficulty.

Yours truly, E. E. P.

The case here presented is certainly a singular and a very unusual one, and especially in the fact that it occurred without any apparent cause or disturbance. In the absence of a personal examination it is not easy to decide as to the best method of treating the case. From what is given in this description one or two things may be suggested, and the first thought is, that an appliance might be adapted with a view of drawing the tooth down to its proper position; this probably would be easily done, but the question arises would it remain? This can only be determined by actual experiment, and if success was attained, it would be by long retention of the tooth in its new position. As to how far, if at all, irritation would be produced by this method can not be decided short of the demonstration. There is some probability that by drawing it down and retaining it firmly in place it would within a few months become fixed and remain.

It is questionable whether in moving the tooth down, the margin of the gum would follow; if this should not occur and the neck of the tooth should be exposed by the elongation, it might be better to adopt some other treatment.

And this might consist in the dressing off of the point of the right central incisor as much as admissible, say a third to half a line, and then lengthen the point of the short tooth by building on gold till it was of the proper length. This method would be less likely to produce disturbance in the socket of the tooth, but the appearance would not be so good as that attained by the first method suggested. Another method, but of more doubtful propriety than either of the others, is to press the right central up into its socket about as far as the other and retain it there till it became fixed; or push it up a little and draw the other down a little. Great caution should be exercised, and close watch should be kept on the case if either of these methods should be adopted. These teeth all have living pulps and these ought not to be destroyed.

## Figures and Tables

**Figure f1:**